# Alterations in energy production in a *Drosophila* model for the X-linked dystonia-parkinsonism-related Taf1 deficiency

**DOI:** 10.3389/fnagi.2026.1684267

**Published:** 2026-02-16

**Authors:** Frida Mandik, Shela Marie Algodon, Philip Seibler, Christine Klein, Melissa Vos

**Affiliations:** Institute of Neurogenetics, University of Luebeck, UKSH, Luebeck, Germany

**Keywords:** *Drosophila melanogaster*, fatty acid oxidation, intellectual disabilities (ID), TAF1, XDP

## Abstract

**Background:**

X-linked dystonia-parkinsonism (XDP), an adult-onset neurodegenerative disorder, is caused by an SVA insertion in the *TAF1* gene, containing a hexanucleotide, the length of which is correlated to the severity of the disease. The SVA insertion moderately disrupts gene expression; however, the underlying disease mechanism remains enigmatic.

**Methods:**

Here, we characterized a fly model for Taf1 deficiency and performed a pilot RNA sequencing analysis. Subsequently, we validated these findings in Taf1-deficient flies and in XDP patient-derived fibroblasts.

**Results:**

We identified an upregulation of genes involved in lipid-dependent energy production as a compensatory mechanism to maintain proper ATP levels. However, studies in XDP patient-derived fibroblasts with minor TAF1 reduction did not confirm these findings.

**Conclusion:**

*β*-oxidation is elevated in flies with severe TAF1 reduction but not detected in XDP-patient fibroblasts, suggesting that this compensatory mechanism may only manifest above a critical TAF1 dosage threshold, absent in patient basal conditions. This finding thus suggests that dosage-dependent metabolic responses occur following TAF1 loss.

## Introduction

X-linked dystonia-parkinsonism (XDP) is a neurodegenerative disorder that presents with focal dystonia, typically manifesting in the fourth decade and evolving to generalized dystonia with parkinsonism, which later becomes the dominant feature (Pauly et al., 2020; Pozojevic et al., 2022). The disease is caused by a retrotransposon insertion consisting of a short interspersed nuclear element (SINE) domain, a variable number of tandem repeats (VNTR), and an Alu-like domain (ALU), commonly referred to as an SVA insertion (Makino et al., 2007). A polymorphic hexanucleotide sequence (AGAGGG)_n_ within the SVA insertion modifies the age at onset with the repeat number being inversely correlated with the age at onset of the disease (Westenberger et al., 2019; Laabs et al., 2021; Trinh et al., 2023) and TAF1 expression (Makino et al., 2007; Domingo et al., 2016; Ito et al., 2016; Bragg et al., 2017; Rakovic et al., 2018; Westenberger et al., 2019), underscoring the contribution of reduced TAF1 levels to the disease. However, the underlying mechanism remains to be elucidated. For this, animal models can be of great value; however, few animal models exist to study XDP (Gudmundsson et al., 2019; Janakiraman et al., 2019), and little is known about the cellular effects caused by TAF1 reduction. Here, we took advantage of *Drosophila melanogaster* as an animal model that has been proven to provide multiple essential insights into various neurodegenerative diseases (Vos and Klein, 2021). We aimed to characterize the effect of loss of Taf1 in flies using an available *taf1*-mutant fruit fly line and performed a pilot RNA sequencing analysis. We validated these findings in *taf1*-mutant flies and used fibroblasts derived from XDP patients to test the relevance of these findings for XDP patients.

## Methods

### Fly genetics

We purchased *taf1^1^red^1^e^1^/TM3, SB^1^*, control (w^1118^), w1118; Mi{ET1}Acadvl^MB08844^, and y1w*; Mi{MIC}CG3902^MI11047^/TM3, Sb1Ser1 lines from the Bloomington Drosophila Stock Center. *Taf1*-mutant flies were crossed to control flies and tested in a heterozygous condition (*w^1118^; taf1^1^/+*).

### RNA analyses

RNA was isolated from 10 whole flies using a standard procedure with the Qiagen RNA isolation kit. RNA sequencing and analysis of the fastq files were performed by the Genomics Core at UZ Leuven (Belgium) on an Illumina HiSeq system. Data analysis was performed using the DAVID Ontology Software.^1^ The data were validated using quantitative real-time PCR (qPCR), for which RNA was isolated from 20 flies or pellets containing approximately 500,000 fibroblasts, which were cultivated overnight at 37 °C using the Monarch Total RNA Miniprep Kit. qPCR was performed using the Maxima SYBR Green/Fluorescein qPCR Master Mix, and the reference genes *dRpl32*, *deEF1a2*, and *dAct5c* for flies and *UBE2D2*, *HPRT*, and *YWHAZ* for fibroblasts were used (Supplementary Table S1). The data for the different references were merged, and the mean value was calculated. The patient-derived fibroblast cultures were derived from four different XDP patients with SVA repeat numbers ranging from 39 to 45 years. Four healthy control fibroblasts with the same ethnicity, age, and sex were also used, as well as four different TAF1-ID patients with missense mutations and their corresponding controls.

### ATP levels

ATP levels were assessed using the ATP Bioluminescence Assay Kit CLS II following the manufacturer’s protocol. Each sample contained two flies, and the bioluminescence was measured using a Synergy HT Multi-Mode Microplate Reader (Biotek). The samples were normalized to the protein concentration using the Pierce BCA Protein Assay Kit.

### Statistical analyses

Nonparametric analyses were performed for experiments with a sample size greater than 7, using GraphPad Prism. The Kruskal–Wallis test was performed for group comparison. For pairwise comparison, the Mann–Whitney *U* test was performed.

## Results

**Figure 1:**
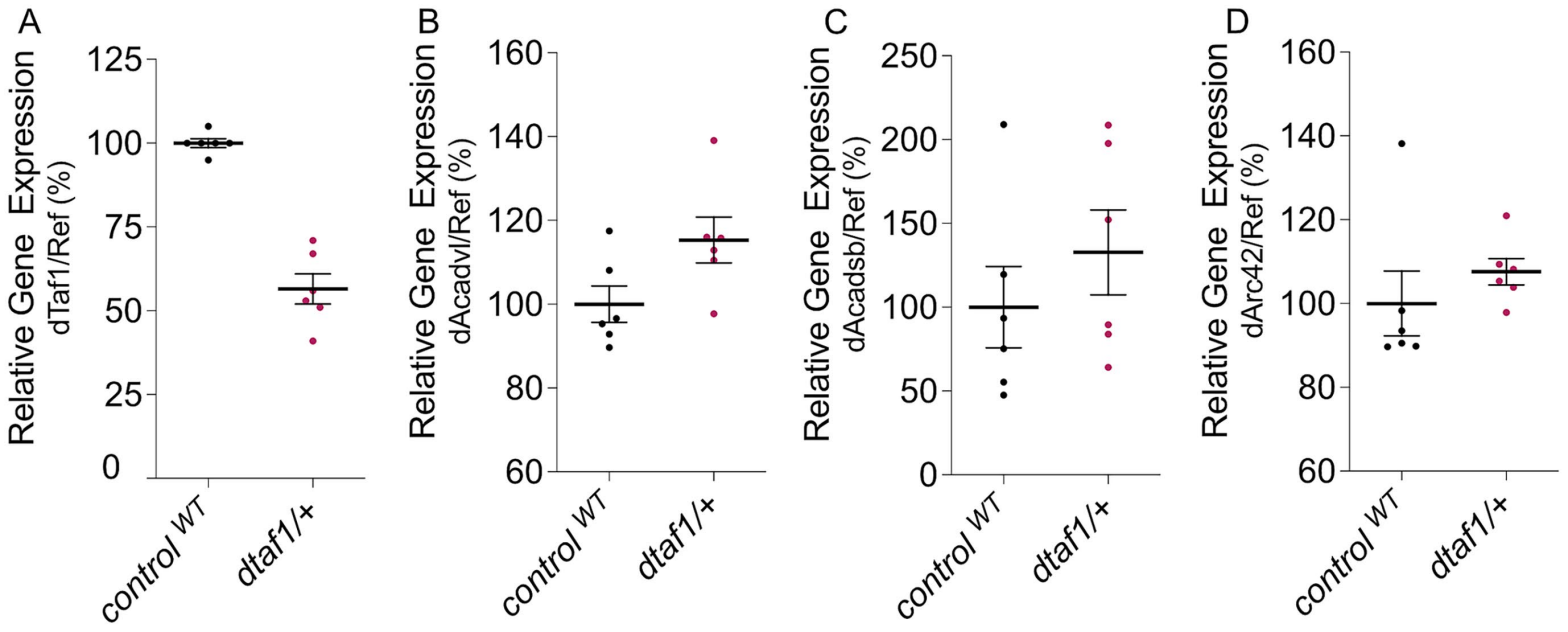
**(A,D)** qPCR data on heterozygous *taf1*-mutant flies compared to control flies to test cDNA levels of *taf1* **(A)**, *Acadvl* **(B)**, *Acadsb* **(C)**, and *Arc42* **(D)**. Data are single percentage data points with means and s.e.m., *n* = 6.

Data from XDP patients reveal a reduction, not a loss, of TAF1 expression levels (Westenberger et al., 2019); hence, we used a heterozygous *taf1*-mutant fly line to mimic these reduced TAF1 levels in patients. Heterozygous loss of Taf1 resulted in a reduction of 57% of *taf1* cDNA compared to control flies (Figure 1A), suggesting these flies are suitable for the study of the effect of reduced TAF1 levels to discover candidate pathways affected by lower TAF1 levels.

The functional impact of TAF1 reduction remains unclear; therefore, we performed a preliminary RNA sequencing analysis comparing the RNA sequencing data of 10 control flies with those of 10 heterozygous *taf1*-mutant flies. KEGG pathway analyses revealed an upregulation of pathways involved in energy production and its different metabolic mechanisms (Supplementary Table S2).

Our data suggest that fatty acid homeostasis is upregulated, contributing to energy production through the degradation of fatty acids in a process known as *β*-oxidation. We focused on this pathway as alterations in *β*-oxidation are common in various neurodegenerative disorders, including Parkinson’s disease (PD) (Vos et al., 2021; Aqeel et al., 2025; Yang et al., 2025), which shares a connection to XDP. To confirm the upregulation of *β*-oxidation upon heterozygous loss of Taf1, qPCR was performed. The first and rate-limiting reaction of *β*-oxidation is catalyzed by Acyl-CoA dehydrogenase (ACAD) (Guerra et al., 2022). Multiple ACADs exist depending on the fatty acids to be processed. We tested the relative expression of ACADs catalyzing very long fatty acids (Acadvl), short fatty acids (Arc42), and branched fatty acids (Acadsb). All three Acads exhibit a tendency towards higher gene expression in heterozygous *taf1*-mutant flies compared to control flies (Figures 1B–D), confirming the findings of our RNA sequencing analysis that *β*-oxidation is increased upon reduction of Taf1.

**Figure 2:**
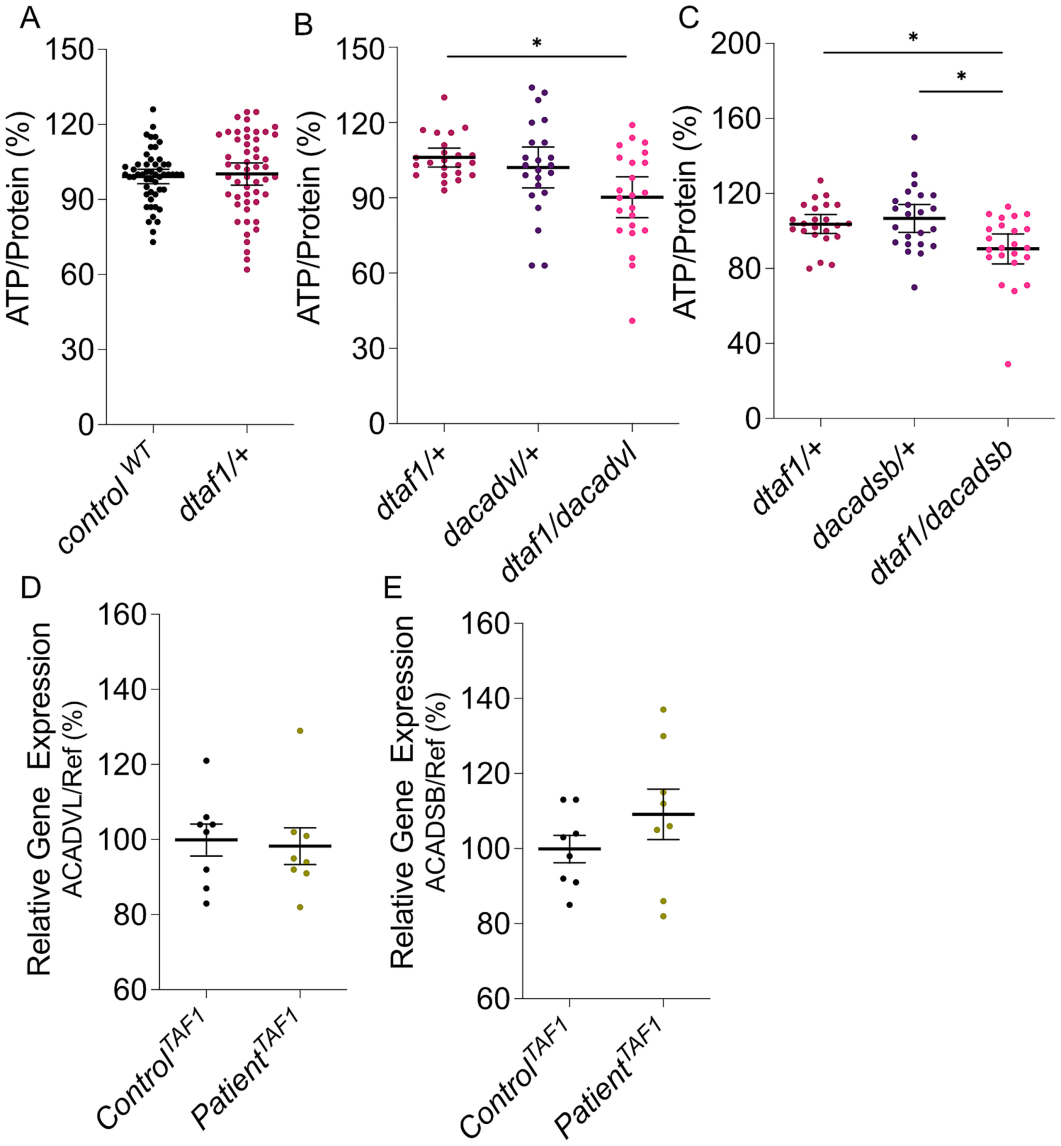
**(A–C)** ATP levels of Taf1-deficient flies compared to control flies **(A)**, *taf1*, *Acadvl,* and double heterozygous mutants *taf1/Acadvl* **(B)**, and *taf1*, *Acadsb,* and double heterozygous mutants *taf1/Acadsb* **(C)**. **(D,E)** qPCR data on XDP patients compared to controls to test cDNA levels of ACADVL **(D)** and ACADSB **(E)**. Data are normalized data points with 95% confidence interval *n* = 23–52 **(A–C)** and single percentage data points with means and s.e.m., *n* = 8 **(D, E)**. The Kruskal–Wallis test was used for group comparison, and the Mann–Whitney U-test was employed for pairwise comparison. *p < 0.05.

In addition to *β*-oxidation, glycolysis appears to be upregulated upon Taf1 deficiency (Supplementary Table S2). Both feed acetyl-CoA into the tricarboxylic acid (TCA) cycle, providing substrates for the mitochondrial electron transport chain, which facilitates oxidative phosphorylation and the production of ATP (Shi and Tu, 2015). Hence, we tested ATP levels, and in *taf1*-mutant flies, these ATP levels are unaffected compared to control flies (Figure 2A), suggesting the upregulation of Acads is a compensatory mechanism to maintain stable ATP levels in Taf1-deficient flies. To assess this, we measured ATP levels in double heterozygous mutant flies carrying a heterozygous loss of Taf1 and Acad. Heterozygous loss of Acad does not affect ATP levels; however, the double heterozygous mutant *taf1*-*Acad* flies have decreased ATP levels (Figures 2B,C), showing that the upregulation of *β*-oxidation upon Taf1 reduction serves as a compensation to stabilize ATP levels following lower Taf1 levels.

To test whether these mechanisms are relevant for XDP patients, we performed qPCR in patient-derived fibroblasts and assessed the expression levels of the different ACADs. No effect was observed in cDNA levels of two different ACADs in patient cells compared to control fibroblasts (Figures 2D,E). Similarly, we did not observe an effect in expression levels of the different ACADs in fibroblasts from TAF1-dependent intellectual disability (TAF1-ID) patients (Supplementary Figure S1), which is caused by missense mutations in *TAF1* and does not result in lower TAF1 expression levels.

## Discussion

TAF1 is required for the proper function of RNA polymerase II and is linked to XDP via an SVA insertion that reduces TAF1 expression levels. Our data support the upregulation of *β*-oxidation due to the heterozygous loss of Taf1 in fruit flies. Furthermore, we discovered that increased *β*-oxidation functions as a compensatory mechanism to maintain stable ATP levels. Nonetheless, we could not confirm this in fibroblasts derived from XDP patients, suggesting that differential processes may exist in flies and humans.

In our pilot RNA sequencing analysis, we obtained mean RNA expression levels for 10 whole flies, providing us with preliminary data that showed the upregulation of *β*-oxidation. Although our sample size is relatively modest, recent studies indicate that this limitation affects statistical power rather than precision (Degen and Medo, 2025). Nonetheless, previous studies in different model organisms have not highlighted pathways involved in energy metabolism as being altered (Domingo et al., 2016; Gudmundsson et al., 2019).

Our results revealed an upregulation of *β*-oxidation and glycolysis, both of which provide substrates for mitochondrial energy production (Shi and Tu, 2015). Interestingly, impaired mitochondrial energy production is a common feature upon neurodegeneration, specifically also for PD (Morais et al., 2009; Vos et al., 2012, 2013), which shares features with late-stage Parkinsonism of XDP. However, in PINK1-related PD, *β*-oxidation levels are decreased, resulting in lower ATP levels (Vos et al., 2021). In contrast, following Taf1 reduction, we observed an upregulation of *β*-oxidation to ensure sufficient ATP levels. Thus, heterozygous loss of Taf1 induces a shift in energy production towards increased *β*-oxidation.

We did not observe the same findings in fibroblasts derived from XDP patients, suggesting that upregulation of *β*-oxidation plays a minor role, if any, in these patients. One possible explanation could lie in the amount of Taf1 reduction in flies compared to that in XDP patients, where the decrease in TAF1 expression is relatively minor (Domingo et al., 2016; Rakovic et al., 2018). This supports that our findings likely reveal a threshold-dependent activation of metabolic compensation, such that at mild reductions, as under (patho)physiological conditions in patients, cells may maintain homeostasis through subtle, sustained adjustments without triggering acute metabolic reprogramming. However, at a more substantial reduction, as observed in our heterozygous *taf1*-mutant flies, transcriptional stress and energy demand may exceed a critical threshold, stimulating cells to activate compensatory pathways, such as enhanced *β*-oxidation, to sustain ATP production. Nonetheless, understanding this compensation is biologically valuable, as identifying the energetic stress pathways triggered by TAF1 loss could reveal cellular vulnerabilities and adaptive limits relevant to XDP pathophysiology. Even if not prominent under basal conditions, *β*-oxidation upregulation may become relevant during metabolic stress, aging, or in response to environmental challenges. In addition to dosage effects, cell-intrinsic metabolic specialization likely contributes to the discrepancy between the findings in fly and patient fibroblasts. Neurons, particularly those affected in XDP (dopaminergic and medium spiny neurons), depend almost exclusively on oxidative phosphorylation (~95% ATP production), whereas fibroblasts predominantly employ aerobic glycolysis with relatively modest mitochondrial capacity. Consequently, TAF1 reduction will primarily compromise neuronal oxidative phosphorylation capacity, creating an energy deficit that may be compensated for by upregulating *β*-oxidation, as we observe in our fly data. Fibroblasts, by contrast, can maintain ATP homeostasis through residual glycolytic capacity without requiring metabolic compensation. An additional consideration is that TAF1 undergoes tissue-specific splicing, and neuronal-specific isoforms may differentially regulate genes controlling mitochondrial biogenesis and energy metabolism. Finally, our RT-qPCR analysis was limited to human orthologs of fly genes showing the most significant fold changes in *β*-oxidation. A more comprehensive transcriptomic profiling in patient-derived neural cells would be essential to determine whether elevated *β*-oxidation occurs in XDP patient neurons and, if so, whether this represents a protective maladaptive response.

## Conclusion

Little is known about the effects of *TAF1* mutations in humans and their role in various diseases. Therefore, characterizing these effects in animal models may provide valuable insights into TAF1-related mechanisms leading to disease. Our study identified significant disruptions in energy production in Taf1-deficient flies. Although we could not confirm these findings in fibroblasts derived from XDP patients, these mechanisms may still be relevant in TAF1 deficiency. Still, rather than being a primary disease driver in XDP patients with milder TAF1 reduction, they may be a threshold-dependent compensatory response.

## Data Availability

The data has been uploaded to Gene Expression Omnibus (GEO) and is available under the accession ID GSE318289 and is available via https://www.ncbi.nlm.nih.gov/geo/query/acc.cgi?acc=GSE318289.

## Footnotes

1
https://davidbioinformatics.nih.gov/home.jsp
