# Supplementary material for: Alterations in energy production in a Drosophila model for the X-linked dystonia-parkinsonism-related Taf1 deficiency
Source: Front Aging Neurosci. 2026 Feb 16;18:1684267. doi: 10.3389/fnagi.2026.1684267 (PMC12950743; doi:10.3389/fnagi.2026.1684267)
Supplement: Supplementary file 1 [file Data_Sheet_1.docx]

**Supplemental Fig. 1.** (A-B) Gene expression data on TAF1-ID patients compared to controls to test cDNA levels of *ACADVL* (A) and *ACADSB* (B). Data are single percentage data points with means and s.e.m.; n = 12. The Whitney U-test was employed for pairwise comparison.
