# Supplementary material for: Alterations in energy production in a Drosophila model for the X-linked dystonia-parkinsonism-related Taf1 deficiency
Source: Front Aging Neurosci. 2026 Feb 16;18:1684267. doi: 10.3389/fnagi.2026.1684267 (PMC12950743; doi:10.3389/fnagi.2026.1684267)
Supplement: Supplementary file 2 [file Table_1.docx]

Supplemental Table 1. Primer used in the qPCR

| Target Gene | Primer Orientation | Sequence | FlyBase ID/ OMIM |
| --- | --- | --- | --- |
| *ACADSB* | Forward | CAGATGCTGGGACTGGCG | 600301 |
|  | Reverse | TGGCCATTGACGCTTCTTTT |  |
| *ACADVL* | Forward | GCCATCGACCTCTATGCCAT | 609575 |
|  | Reverse | CAAGTGGGTTGCTGGTGAC |  |
| *dAcadsb* | Forward | CTCTGATGTGGCGGGAGTCT | FBgn0036824 |
|  | Reverse | CTTGCGCTCCAGCAGGTAC |  |
| *dAcadvl* | Forward | GTGGCCACGTAGTGGGTGA | FBgn0034432 |
|  | Reverse | GTGGTCTTGGCGATGGTGG |  |
| *dAct5c* | Forward | TCCACGAGACCACCTACAAC | FBgn0000042 |
|  | Reverse | CACTTGCGGTGCACAATGGA |  |
| *dArc42* | Forward | CCACCATAGCAACCGACAAG | FBgn0038742 |
|  | Reverse | GATGCCCAGCTTATCCTCCT |  |
| *deEF1a2* | Forward | GCGTGGGTTTGTGATCAGTT | FBgn0000557 |
|  | Reverse | GATCTTCTCCTTGCCCATCC |  |
| *dRPL32* | Forward | ATCGGTTACGGATCGAACAA | FBgn0002626 |
|  | Reverse | GACAATCTCCTTGCGCTTCT |  |
| *dTaf1* | Forward | CCCAACTACGATCCCTCAGA | FBgn0010355 |
|  | Reverse | CTCGAGAACCATCGTCTTCC |  |
| *HPRT1* | Forward | ATCAGACTGAAGAGCTATTGTAATGACCA | 308000 |
|  | Reverse | TGGCTTATATCCAACACTTCGTG |  |
| *UBE2D2* | Forward | TGCCTGAGATTGCTCGGATCT | 602962 |
|  | Reverse | TCGCATACTTCTGAGTCCATTCC |  |
| *YWHAZ* | Forward | GTAGGTCATCTTGGAGGGTCGT | 601288 |
|  | Reverse | GGTATGCTTGTTGTGACTGATCG |  |
