# Supplementary material for: Alterations in energy production in a Drosophila model for the X-linked dystonia-parkinsonism-related Taf1 deficiency
Source: Front Aging Neurosci. 2026 Feb 16;18:1684267. doi: 10.3389/fnagi.2026.1684267 (PMC12950743; doi:10.3389/fnagi.2026.1684267)
Supplement: Supplementary file 3 [file Table_2.docx]

Supplemental Table 2. KEGG Pathways that were significantly upregulated or downregulated in heterozygous taf1-mutant flies ranked according to p-value

| UPREGULATED | | | |  | | |  |
| --- | --- | --- | --- | --- | --- | --- | --- |
| Term | Count | p-value | | FDR (Benjamini-Hochberg) | |  |  |
| dme01100:Metabolic pathways | 324 | 2.2E-36 | | 3.05E-34 | |  |  |
| dme00190:Oxidative phosphorylation | 52 | 2.0E-8 | | 1.36E-06 | |  |  |
| dme04142:Lysosome | 46 | 3.8E-8 | | 1.56E-06 | |  |  |
| dme00980:Metabolism of xenobiotics by cytochrome P450 | 31 | 5.1E-8 | | 1.56E-06 | |  |  |
| dme00983:Drug metabolism - other enzymes | 38 | 5.7E-8 | | 1.56E-06 | |  |  |
| dme00982:Drug metabolism - cytochrome P450 | 29 | 3.9E-7 | | 9.07E-06 | |  |  |
| dme00040:Pentose and glucuronate interconversions | 24 | 7.8E-7 | | 1.53E-05 | |  |  |
| dme00500:Starch and sucrose metabolism | 17 | 1.0E-5 | | 1.78E-04 | |  |  |
| dme00053:Ascorbate and aldarate metabolism | 17 | 4.3E-5 | | 6.56E-04 | |  |  |
| dme00280:Valine, leucine and isoleucine degradation | 16 | 5.2E-5 | | 7.16E-04 | |  |  |
| dme01212:Fatty acid metabolism | 21 | 1.4E-4 | | 1.73E-03 | |  |  |
| dme01200:Carbon metabolism | 36 | 3.2E-4 | | 3.64E-03 | |  |  |
| dme00071:Fatty acid degradation | 15 | 3.4E-4 | | 3.64E-03 | |  |  |
| dme01240:Biosynthesis of cofactors | 39 | 5.3E-4 | | 5.22E-03 | |  |  |
| dme00232:Caffeine metabolism | 6 | 6.1E-4 | | 5.60E-03 | |  |  |
| dme00830:Retinol metabolism | 14 | 1.3E-3 | | 1.05E-02 | |  |  |
| dme00630:Glyoxylate and dicarboxylate metabolism | 14 | 1.3E-3 | | 1.05E-02 | |  |  |
| dme01230:Biosynthesis of amino acids | 22 | 2.1E-3 | | 1.58E-02 | |  |  |
| dme00790:Folate biosynthesis | 15 | 3.2E-3 | | 2.30E-02 | |  |  |
| dme00350:Tyrosine metabolism | 10 | 5.4E-4 | | 3.71E-02 | |  |  |
| dme00480:Glutathione metabolism | 23 | 6.7E-3 | | 4.38E-02 | |  |  |
| dme00052:Galactose metabolism | 13 | 7.4E-4 | | 4.65E-02 | |  |  |
| dme00640:Propanoate metabolism | 11 | 7.7E-3 | | 4.65E-02 | |  |  |
| dme00270:Cysteine and methionine metabolism | 13 | 1.2E-2 | | 6.92E-02 | |  |  |
| dme00561:Glycerolipid metabolism | 14 | 1.4E-2 | | 7.14E-02 | |  |  |
| dme00410:Beta-Alanine metabolism | 10 | 1.4E-2 | | 7.14E-02 | |  |  |
| dme00730:Thiamine metabolism | 9 | 1.4E-2 | | 7.14E-02 | |  |  |
| dme00860:Porphyrin metabolism | 15 | 1.5E-2 | | 7.59E-02 | |  |  |
| dme04146:Peroxisome | 24 | 1.9E-2 | | 8.68E-02 | |  |  |
| dme00511:Other glycan degradation | 9 | 1.9E-2 | | 8.68E-02 | |  |  |
| dme00620:Pyruvate metabolism | 15 | 2.2E-2 | | 9.89E-02 | |  |  |
| dme00260:Glycine, serine and threonine metabolism | 10 | 2.4E-2 | | 1.02E-01 | |  |  |
| dme00531:Glycosaminoglycan degradation | 7 | 2.5E-2 | | 1.06E-01 | |  |  |
| dme00010:Glycolysis / Gluconeogenesis | 16 | 2.8E-2 | | 1.12E-01 | |  |  |
| dme00670:One carbon pool by folate | 6 | 3.4E-2 | | 1.34E-01 | |  |  |
| dme04080:Neuroactive ligand-receptor interaction | 18 | 3.9E-2 | | 1.46E-01 | |  |  |
| dme00020:Citrate cycle (TCA cycle) | 13 | 3.9E-2 | | 1.46E-01 | |  |  |
| dme00520:Amino sugar and nucleotide sugar metabolism | 14 | 4.1E-2 | | 1.49E-01 | |  |  |
| dme00230:Purine metabolism | 23 | 4.3E-2 | | 1.52E-01 | |  |  |
| dme00061:Fatty acid biosynthesis | 6 | 4.8E-2 | | 1.65E-01 | |  |  |
| DOWNREGULATED | |  | |  | |  |  |
| Term | Count | | p-value | | FDR (Benjamini-Hochberg) |  |  |
| dme04141:Protein processing in endoplasmic reticulum | 35 | | 0.0015 | | 0.17 |  |  |
| dme04068:FoxO signaling pathway | 18 | | 0.0028 | | 0.15 |  |  |
| dme04711:Circadian rhythm - fly | 6 | | 0.0084 | | 0.28 |  |  |
| dme03030:DNA replication | 12 | | 0.0208 | | 0.46 |  |  |
| dme00510:N-Glycan biosynthesis | 12 | | 0.0257 | | 0.45 |  |  |
| dme04120:Ubiquitin mediated proteolysis | 24 | | 0.0439 | | 0.58 |  |  |
| dme04013:MAPK signaling pathway - fly | 7 | | 0.0477 | | 0.56 |  |  |
